# Supplementary material for: Triacylglycerol-droplet-induced bilayer spontaneous curvature in giant unilamellar vesicles
Source: Biophys J. 2024 May 31;123(13):1857–68. doi: 10.1016/j.bpj.2024.05.030 (PMC11267425; doi:10.1016/j.bpj.2024.05.030)
Supplement: Document S1. Tables S1 and S2 [file mmc1.pdf]

Biophysical Journal, Volume *123*

Supplemental information

Triacylglycerol-droplet-induced bilayer spontaneous curvature in giant unilamellar vesicles

Chiho Kataoka-Hamai

Table S1. Vesicle radii (R_v) of unilamellar vesicles incorporating TAG droplets.

PC	TAG	R_v (± standard error) (μm)	
18:1	14:1	3.79 (± 0.26)	(N = 30)
	16:1	3.50 (± 0.18)	(N = 18)
	18:1	3.63 (± 0.23)	(N = 19)
	20:1	3.76 (± 0.23)	(N = 24)
16:0–18:1	14:1	3.31 (± 0.22)	(N = 19)
	16:1	3.11 (± 0.23)	(N = 23)
	18:1	3.36 (± 0.31)	(N = 21)
	20:1	4.02 (± 0.66)	(N = 9)
16:1	14:1	3.16 (± 0.12)	(N = 35)
	16:1	3.12 (± 0.14)	(N = 23)
	18:1	2.80 (± 0.24)	(N = 16)
	20:1	2.75 (± 0.16)	(N = 31)
14:1	14:1	4.28 (± 0.33)	(N = 31)
	16:1	3.82 (± 0.60)	(N = 11)
	18:1	4.79 (± 0.37)	(N = 13)
	20:1	4.22 (± 0.36)	(N = 9)

Table S2. Size parameters (R_0) of the vesicles consisting of single-bilayer and double-bilayer segments. The R_0 values were calculated by assuming that the vesicles had a total bilayer surface area of $4\pi R_0^2$.

PC	TAG	R_0 (± standard error) (μm)	
18:1	14:1	5.82 (± 0.85)	(N = 4)
	16:1	5.53 (± 0.50)	(N = 14)
	18:1	5.99 (± 0.45)	(N = 17)
	20:1	7.27 (± 0.58)	(N = 16)
16:0–18:1	14:1	6.01 (± 0.63)	(N = 13)
	16:1	6.40 (± 0.72)	(N = 15)
	18:1	3.56	(N = 1)
	20:1	5.25 (± 0.57)	(N = 7)
16:1	14:1	3.62 (± 0.07)	(N = 2)
	16:1	4.90 (± 0.34)	(N = 21)
	18:1	5.42 (± 0.41)	(N = 12)
	20:1	4.31 (± 0.30)	(N = 19)
14:1	14:1	5.08 (± 0.80)	(N = 4)
	16:1	5.39 (± 0.32)	(N = 18)
	18:1	7.54 (± 0.47)	(N = 39)
	20:1	5.49 (± 0.34)	(N = 20)
