# Supplementary material for: Triacylglycerol-droplet-induced bilayer spontaneous curvature in giant unilamellar vesicles
Source: Biophys J. 2024 May 31;123(13):1857–68. doi: 10.1016/j.bpj.2024.05.030 (PMC11267425; doi:10.1016/j.bpj.2024.05.030)
Supplement: Document S2. Article plus supporting material [file mmc2.pdf]

Biophysical Journal
Article

Triacylglycerol-droplet-induced bilayer spontaneous curvature in giant unilamellar vesicles

Chiho Kataoka-Hamai[1,*]
[1]Research Center for Macromolecules and Biomaterials, National Institute for Materials Science, Tsukuba, Ibaraki, Japan

ABSTRACT This study investigated the incorporation of triacylglycerol droplets in the bilayers of giant unilamellar vesicles (GUVs) using four triacylglycerols and four phosphatidylcholines by confocal laser scanning microscopy. The triacylglycerol droplets were incorporated between the monolayer leaflets of the GUVs. Among the spherical droplets protruding on only one side of the bilayers, the droplets bound to the outer leaflets outnumbered those bound to the inner leaflets. The more frequent droplet binding to the outer leaflet caused transbilayer asymmetry in the droplet surface density. A vesicle consisting of a single-bilayer spherical segment and a double-bilayer spherical segment was also observed. The yield of these vesicles was comparable with or higher than that of the droplet-incorporating GUVs for many of the phosphatidylcholine-triacylglycerol combinations. In a vesicle consisting of single-bilayer and double-bilayer segments, most of the triacylglycerol droplets were localized on the outermost membrane surface along the segment boundary and in the double-bilayer segment. To rationalize the formation of these vesicle structures, we propose that the transbilayer asymmetry in the droplet surface density induces spontaneous curvature of the bilayer, with the bilayer spontaneously bending away from the droplets. Energy calculations performed assuming the existence of spontaneous curvature of the bilayer corroborated the experimentally determined membrane shapes for the vesicles consisting of unilamellar and bilamellar regions.

SIGNIFICANCE Phospholipid bilayers have energetically preferred curvature, which is referred to as bilayer spontaneous curvature. Bilayer spontaneous curvature regulates the membrane shape and affects cellular processes. The roles of the phospholipid composition, sugars, ions, polymers, and proteins in bilayer spontaneous curvature have been investigated. However, the interplay between bilayer spontaneous curvature and fat droplets is not well understood. Fat is stored in lipid droplets, which emerge from the endoplasmic reticulum membrane, consisting of bilayer sheets and tubules with various bilayer curvatures. It is therefore important to decipher the role of bilayer curvature in lipid-droplet biogenesis. In this work, we considered the curvature to understand the interaction between a fat droplet and a phospholipid bilayer.

INTRODUCTION

Lipid droplets are intracellular organelles that are involved in energy production and lipid metabolism (1,2). Their structures consist of a neutral lipid core surrounded by a phospholipid monolayer containing proteins (1–3). The main components of the neutral lipid core are triacylglycerols (TAGs) and cholesteryl esters. To understand the properties and functions of lipid droplets, the basic principles in the interaction between neutral lipids and phospholipids should be understood. Investigation of the interaction mechanisms requires accurate control of multiple parameters, such as the temperature, phospholipid composition, and neutral lipid composition, because the characteristics of phospholipid membranes and neutral lipids vary with these parameters (4,5).

Model membrane systems are useful for dissecting the influence of various parameters. Previous studies have used phospholipid monolayers created at the TAG-water interface to clarify the behavior of TAG molecules in monolayers (6,7). Supported lipid bilayers have also been used to investigate the Ostwald ripening of TAG droplets occurring via TAG diffusion across phospholipid bilayers (8,9). Another useful model system is giant unilamellar vesicles (GUVs). Seminal studies have shown that GUVs easily incorporate TAG droplets upon exposure to a TAG emulsion (10,11). After the incorporation of a TAG droplet in a GUV, the droplet interface is surrounded by phospholipid monolayer leaflets, which seal together around the droplet to create a bilayer structure. This method previously has been used to

Submitted January 16, 2024, and accepted for publication May 29, 2024.
*Correspondence: kataoka.chiho@nims.go.jp
Editor: Rumiana Dimova.
https://doi.org/10.1016/j.bpj.2024.05.030
© 2024 Biophysical Society.
This is an open access article under the CC BY license (http://creativecommons.org/licenses/by/4.0/).

verify the effect of the membrane tension on the direction of droplet budding (10).

In this study, we focused on bilayer spontaneous curvature. When all properties are symmetric across the bilayer midplane, the two monolayer leaflets are indistinguishable; therefore, the bilayer prefers a planar structure. However, when the bilayer consists of asymmetric leaflets across the bilayer midplane, it may bend spontaneously (12). This curvature is referred to as bilayer spontaneous curvature, which occurs because of various transbilayer asymmetries. For example, the attachment of a flexible polymer to one leaflet through a polymer end generates bilayer spontaneous curvature to enhance the entropy of the polymer chain (13). Lateral protein crowding on one leaflet (14) or asymmetric binding of crescent-shaped proteins (15,16) also induce spontaneous curvature. Furthermore, the asymmetric adsorption of monovalent or divalent ions results in spontaneous curvature owing to local phospholipid condensation mediated by the adsorbed ions (17,18). Other examples of transbilayer asymmetry that induce bilayer spontaneous curvature include an asymmetric phospholipid distribution (19), the asymmetric oxidation of unsaturated phospholipids (20), and the inclusion of different sugars across the bilayer (21). Despite the various mechanisms, the induced spontaneous curvature typically leads to a vesicle shape transition, such as tubulation (14,18,19) or vesicle budding (13,20).

Lipid droplets form at the endoplasmic reticulum (ER) membrane (22). TAG is synthesized in the ER bilayer and accumulates to create a lens structure. The TAG lens subsequently grows to bud into the cytosol. The ER is a continuous membrane network consisting of sheets and tubules characterized by different bilayer curvatures (23,24). Thus, understanding the role of bilayer curvature in lipid-droplet biogenesis is important. TAG lenses form at ER tubules (25). A previous study reported that TAG molecules more readily assemble to form a cluster in bilayer tubules than in flat bilayers because highly curved bilayers do not stably maintain TAG molecules that freely diffuse in the bilayer (26).

In this study, we investigated the incorporation of TAG droplets in GUV bilayers using confocal laser scanning microscopy. We showed that a vesicle composed of a single-bilayer spherical segment and a double-bilayer spherical segment readily forms after the incubation of GUVs with TAG suspensions. We expect that the formation of these vesicles is due to bilayer spontaneous curvature.

MATERIALS AND METHODS

Materials

1,2-Dioleoyl-*sn*-glycero-3-phosphocholine ((18:1) phosphatidylcholine [PC]) (purity >99%), 1-palmitoyl-2-oleoyl-glycero-3-phosphocholine ((16:0–18:1) PC) (purity > 99%), 1,2-dipalmitoleoyl-*sn*-glycero-3-phosphocholine ((16:1) PC) (purity > 99%), and 1,2-dimyristoleoyl-*sn*-glycero-3-phosphocholine ((14:1) PC) (purity > 99%) were purchased from Avanti Polar Lipids (Alabaster, AL). The concentrations of the PC stock solutions in chloroform were determined by a phosphorus assay (27). Trieicosenoin ((20:1) TAG) (purity > 99%), triolein ((18:1) TAG) (purity > 99%), trihexadecenoin ((16:1) TAG) (purity > 99%), and tritetradecenoin ((14:1) TAG) (purity > 99%) were purchased from Nu-Check Prep (Elysian, MN). A fluorescent probe for the PC bilayers, Texas Red 1,2-dihexadecanoyl-*sn*-glycero-3-phosphoethanolamine, triethylammonium salt (TR-DHPE), was purchased from Thermo Fisher Scientific (Waltham, MA). A fluorescent probe for TAG droplets, 1,2-dioleoyl-3-[11-(dipyrrometheneboron difluoride)undecanoyl]-*sn*-glycerol (TopFluor TG), was purchased from Avanti Polar Lipids. The glass coverslips (25-mm diameter, no. 1) were purchased from Matsunami Glass Ind. (Osaka, Japan).

GUV preparation

The GUVs were prepared by electroformation as follows (28–30). PC was mixed with TR-DHPE (0.2 mol %) in chloroform. The mixture (3.5 mM lipid) with a volume of 4 μL was spread onto two platinum electrodes (0.5-mm diameter) separated by ~1 mm, which were connected to a function generator (PCGU1000, Velleman, Gavere, Belgium). After removing the solvent under a nitrogen stream, the electrodes were immersed in sucrose solution (~300 mM) with a volume of 200 μL. An alternating voltage with a peak-to-peak amplitude of 2.2 V and a frequency of 10 Hz was applied for 30 min. A voltage with the same amplitude and a frequency of 2 Hz was applied for another 5 min.

TAG droplet incorporation in the GUVs

For fluorescence imaging, TAG was mixed with TopFluor TG (0.05 mol %). The labeled TAG sample was suspended by pipetting it in a salt-containing sucrose solution (~40 mM NaCl, ~191 mM sucrose, and ~10 mM NaH_2PO_4-Na_2HPO_4 [pH 7.4]) with a volume of 30–50 μL to give a TAG volume ratio of 1%. The addition of salt was required to enhance the droplet incorporation efficiency in the GUV bilayers. The TAG mixture was sonicated for 10 min in an ultrasonic bath, vortexed, and further sonicated for 10 min to form an emulsion solution. The sonication was performed in a water bath with ice packs to prevent the sample from heating. Immediately after sonication, 5 μL of the emulsion sample was added to the same volume of a GUV solution (0.07 mM phospholipid) and mixed by pipetting slowly four times. The sample was subsequently incubated in the dark for 30 min using a rotating mixer. For fluorescence imaging, the sample (10 μL) was diluted in 40 μL of a glucose solution (~300 mM), followed by deposition onto a glass coverslip in a poly(dimethylsiloxane) chamber. The chamber was sealed with adhesive tape to prevent water evaporation during the observation. The coverslip was fixed in an Attofluor cell chamber (Thermo Fisher Scientific). The final solution consisted of ~49 mM sucrose, ~240 mM glucose, ~4 mM NaCl, and ~1 mM sodium phosphate (pH 7.4). Before sample deposition, the coverslip surface was cleaned with ethanol and dried in a nitrogen stream.

Confocal laser scanning microscopy

Fluorescence imaging was performed with a Zeiss LSM 900 microscope using a 63× oil-immersion objective (Carl Zeiss, Oberkochen, Germany). TR-DHPE and TopFluor TG were excited with 561- and 488-nm lasers, respectively. The fluorescence signals from both dyes were simultaneously recorded in confocal imaging mode using two GaAsP detectors. The fluorescence data were analyzed using ImageJ software (31–33).

Osmolarity measurements

We measured the osmolarities of the sucrose solution used to form the GUVs (~300 mM sucrose) and the sucrose-glucose mixture used to

suspend the GUVs for fluorescence imaging (~49 mM sucrose, ~240 mM glucose, ~4 mM NaCl, and ~1 mM sodium phosphate [pH 7.4]) in milliosmoles per kilogram (mOsm/kg) using a freezing-point osmometer (Osmomat 3000D, ELITechGroup, Puteaux, France) at UBE Scientific Analysis Laboratory (Tokyo, Japan). For the unit conversion to mOsm/L, we determined the densities of the sucrose solution (1.0368 g/mL) and sucrose-glucose mixture (1.0208 g/mL) using a DMA 35 density meter (Anton Paar, Graz, Austria). The osmolarities of the sucrose solution and sucrose-glucose mixture were 358 (±1) and 337 (±1) mOsm/L, respectively ($n = 3$).

Experimental values

All reported values are the mean values (± SE) of n determinations.

RESULTS

TAG droplets incorporated in the GUVs

We used four PCs and four TAGs (Fig. 1) to investigate the incorporation of TAG droplets in the GUVs. After incubation of the GUVs with TAG suspensions, we observed unilamellar vesicles with TAG droplets (Fig. 2 *A*). The radius of the GUVs was approximately 4 μm (Table S1). The TopFluor TG fluorescence signals from the droplets (*green*) were surrounded by the TR-DHPE fluorescence signals from the membranes (*red*). Previous studies have reported that such droplets are sandwiched between the monolayer leaflets of the GUVs (34,35). Nonspherical droplets (Fig. 2 *A*(*i*) and some spherical droplets (Fig. 2 *A*(*ii*)) protruded both outside and inside the vesicles. Other spherical droplets bound to the outer (Fig. 2 *A*(*iii*)) or inner (Fig. 2 *A*(*iv*)) leaflet.

We investigated the fractions of the spherical droplets bound to the outer leaflets (Fig. 2 *A*(*iii*)) and those bound to the inner leaflets (Fig. 2 *A*(*iv*)) among all observed droplets embedded in the GUV bilayers. The results (Fig. 2 *B*) showed that for most PC-TAG combinations, the fraction of the droplets bound to the outer leaflets (*gray*) was high, whereas the fraction of those bound to the inner leaflets (*black*) was very low. The more frequent droplet binding to the outer leaflets implies that GUVs often had an asymmetric droplet surface density across the bilayer membranes.

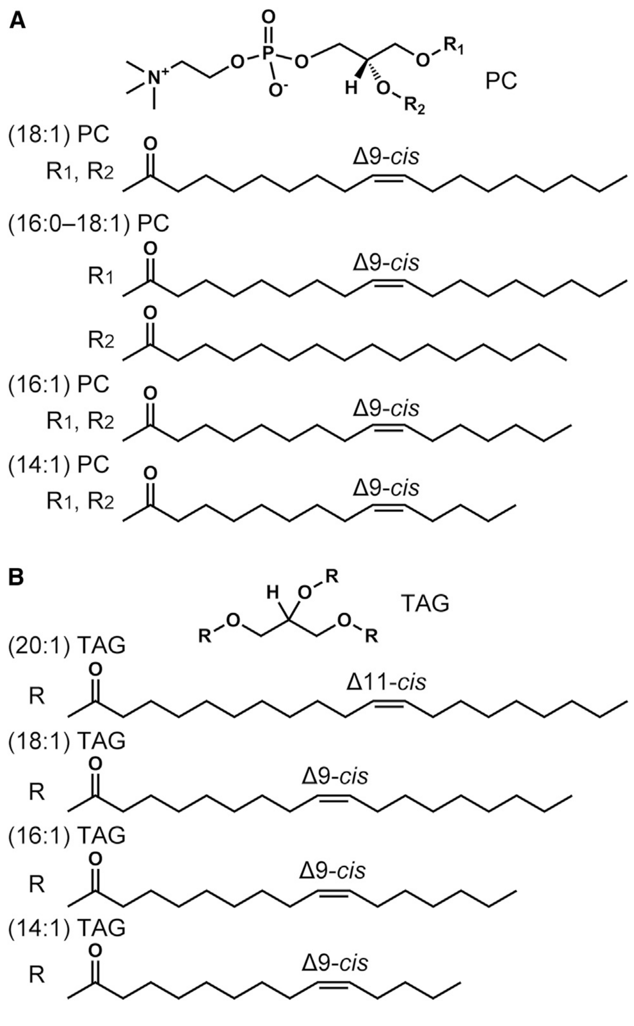

FIGURE 1 Chemical structures of the (*A*) PCs and (*B*) TAGs used in this study.

Formation of vesicles consisting of a single-bilayer region and a double-bilayer region

In addition to droplet-incorporating unilamellar vesicles, we often observed vesicles consisting of two spherical segments with high and low TR fluorescence intensities (Fig. 3 *A*). The plane of the intersection of the spherical segments was devoid of membrane structures (Fig. 3 *B*). Such vesicles were not observed in the GUV preparations without TAGs, suggesting that TAGs altered the membrane structures. We investigated the shapes of these vesicles using the intersection angle (θ) between the tangent planes to the spherical segments at a point of intersection of the two membrane segments (Fig. 3 *C*). We attributed the segments of high and low TR fluorescence emissions to double-bilayer and single-bilayer regions, respectively, as described later. We visualized vesicles with various θ values (Fig. 3 *D*). The largest value was 180° (Fig. 3 *D*(*v*)), which characterizes a round vesicle consisting of two concentric segments, as depicted in Fig. 3 *E*(*ii*).

We ascribed the spherical segments to a single-bilayer membrane and a double-bilayer membrane based on the following results. First, the TR fluorescence intensities in the low and high TR fluorescence regions were similar to, and approximately two times larger than, those in unilamellar vesicle bilayers, respectively. The image slice in Fig. 3 *F*(*i*) shows a vesicle consisting of two membrane regions (*bottom*) and a unilamellar vesicle (*top right corner*). These vesicles showed the largest profiles on this image slice. We examined the TR fluorescence intensity per pixel for the unilamellar vesicle bilayer enclosed by the blue lines in Fig. 3 *F*(*ii*) (region GUV), as well as those for the membrane

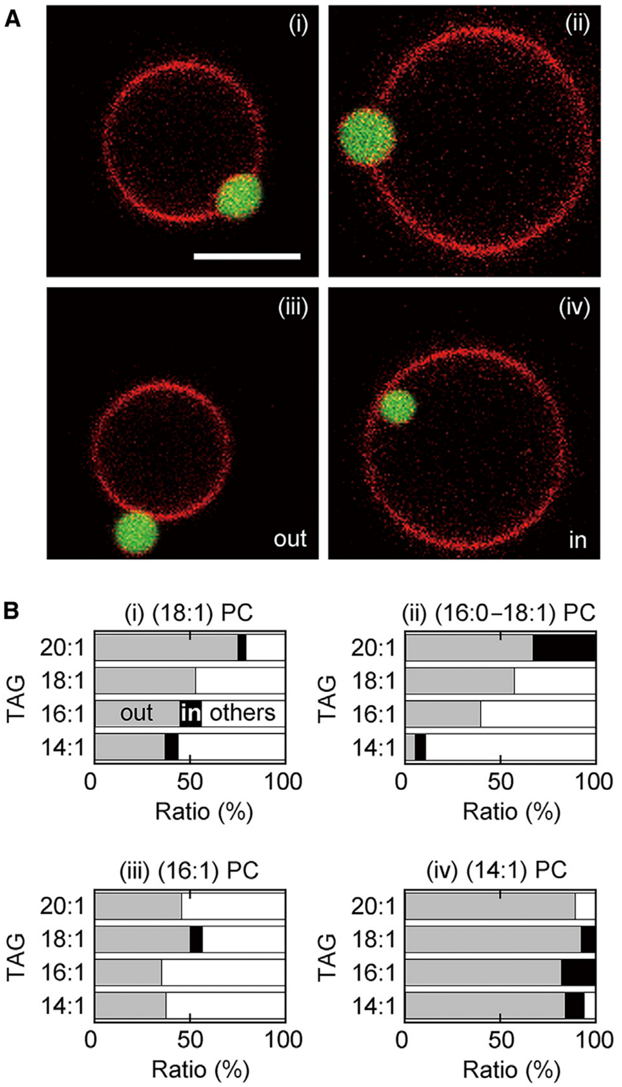

FIGURE 2 Characterization of the TAG droplets incorporated in the GUVs. (*A*) Fluorescence images obtained by simultaneous recording of the TR-DHPE (*red*) and TopFluor TG (*green*) fluorescence signals. The PC-TAG combinations were (*i*) (18:1) PC-(20:1) TAG, (*ii*) (18:1) PC-(18:1) TAG, (*iii*) (14:1) PC-(18:1) TAG, and (*iv*) (14:1) PC-(16:1) TAG. Scale bar, 5 μm (*i*). All images are in the same scale. (*B*) Fractions of spherical TAG droplets bound to the outer leaflets (*gray*, as in *A*(*iii*)), spherical droplets bound to the inner leaflets (*black*, as in *A*(*iv*)), and other remaining droplets (*white*) for various PC-TAG combinations ($n = 9$–35). To see this figure in color, go online.

regions enclosed by the yellow (region DB) and green lines (region SB). The distributions of the TR fluorescence intensity (Fig. 3 *F*(*iii*)) showed that the fluorescence signals from the GUV and SB regions were similar, whereas those from the DB region were nearly twice as high as those from the GUV and SB regions. TR fluorescence signals typically increase proportionally to the number of bilayers (36). Thus, the DB region was seemingly bilamellar. We obtained further evidence for the identification of the membrane segments from the vesicle rupture events (Fig. 3 *G*). When a vesicle bearing high and low TR fluorescence regions (Fig. 3 *G*(*i*)) ruptured on the glass surface, the resultant planar bilayer patch always bound a unilamellar vesicle (Fig. 3, *G*(*ii*) and *H*). From these rupture data, we estimated the total bilayer surface area by assuming that the initial intact vesicle consisted of unilamellar and bilamellar membranes. The total bilayer surface areas before and after rupture were estimated to be 333 and 347 μm^2, respectively. The similar surface areas verified the existence of the single-bilayer and double-bilayer regions in the intact vesicle. We therefore concluded that vesicles with sections of different TR fluorescence intensities consisted of a unilamellar segment and a bilamellar segment. We expected that the single-bilayer and double-bilayer regions were connected, as depicted in Fig. 3 *E*.

Of the 222 vesicles, 99.5, 81.1, and 17.6% had TAG droplets along the region boundary, in the double-bilayer region and single-bilayer region, respectively. In addition, all vesicles that bound droplets on their single-bilayer region had droplets on their double-bilayer region. Therefore, it seemed that the droplets were preferably incorporated along the region boundary and in the double-bilayer region. Droplets in all regions bound to the outermost membrane surfaces and protruded to the external solution. In addition, the droplets were mobile (Fig. 3 *I*).

We examined the yield of vesicles consisting of single-bilayer and double-bilayer membranes by counting the numbers of these vesicles (N_{sd}) and unilamellar vesicles incorporating TAG droplets (N_g) under a microscope (Fig. 3 *J*). For many of the PC-TAG combinations, the yield of vesicles with unilamellar and bilamellar regions ($100 \times N_{sd}/(N_{sd} + N_g)$) was approximately 50% or higher. Thus, these vesicles were often major membrane structures.

Shape characterization of vesicles consisting of single-bilayer and double-bilayer regions

We characterized the vesicle shape (Fig. 4 *A*) using the intersection angle (θ), the surface area of the single-bilayer segment (S_s), and the surface area of the double-bilayer segment (S_d). We first determined the coordinates of the sphere centers (*black points* in Fig. 4 *A*) and the radii of the single-bilayer (R_s) and double-bilayer (R_d) segments using a three-dimensional TR fluorescence image. We subsequently calculated the θ_s, θ_d, and θ values (Fig. 4 *A*(*ii*)). θ is the sum of θ_s and θ_d, which are the angles between the line connecting the two sphere centers and the normal lines to the spheres at a point of intersection of the spherical segments. We then calculated the S_s and S_d values. S_s equals the bilayer surface area in the single-bilayer segment and S_d is half of the bilayer surface area in the double-bilayer segment. The set of $(S_d/S_s)^{1/2}$ and θ values determines the relative vesicle shape (Fig. 4 B(i)). The shapes calculated at several coordinates (*black points*) have the same bilayer surface area. The fluorescence data show the dependence of θ on $(S_d/S_s)^{1/2}$ (Fig. 4 *B*(*ii*)–(*v*)). For each PC, the

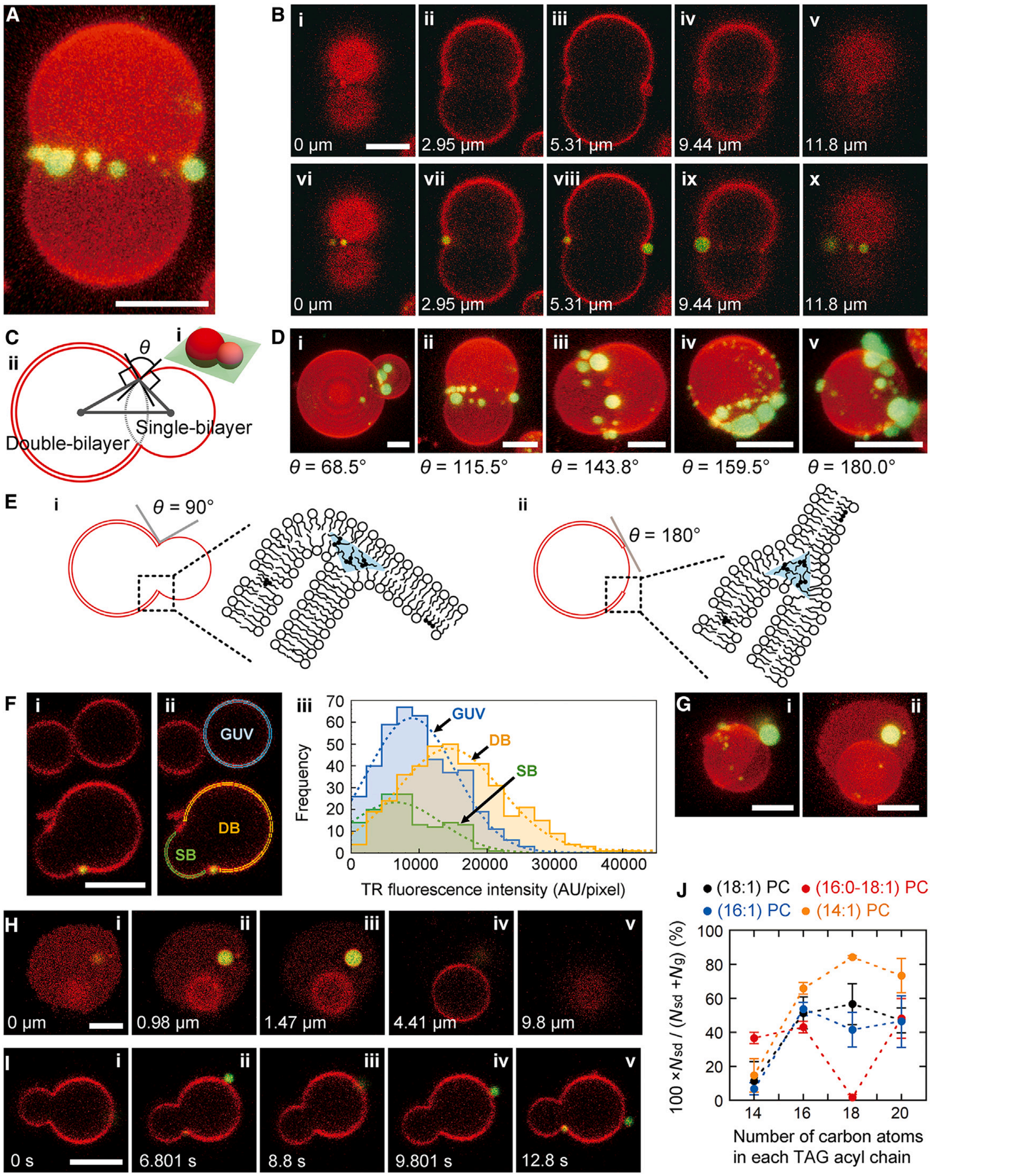

FIGURE 3 Fluorescence data of vesicles consisting of two spherical segments. (*A*, *B*, *D*, and *F–I*) TR-DHPE (*red*) and TopFluor TG (*green*) fluorescence images. Scale bars, 5 μm. (*A*) Three-dimensional image of a (14:1) PC vesicle incubated with (18:1) TAG. The image was constructed from a stack of 25 confocal slices with a separation of 0.59 μm. (*B*) (*i*)–(*v*) TR fluorescence image slices and (*vi*)–(*x*) TR fluorescence image slices combined with the TopFluor fluorescence signals used to construct the three-dimensional image in (*A*). The relative distance from the glass surface is given. (*C*) (*i*) Schematic illustration of a vesicle consisting of single-bilayer and double-bilayer regions, and (*ii*) the cross-sectional view of the green plane in (*i*). The gray points indicate the sphere centers for the membrane segments. The angle, θ, is the angle between the tangents to the two circles at a point of the intersection. (*D*) Fluorescence images of vesicles with various θ values for (*i*) (16:0–18:1) PC-(16:1) TAG, (*ii* and *iii*) (14:1) PC-(18:1) TAG, and (*iv* and *v*) (14:1) PC-(20:1) TAG pairs. The images were constructed from 25 to 27 confocal slices with an interslice distance of 0.35–1.09 μm. (*E*) Schematic illustrations of the intersection regions with (*i*) $\theta = 90°$ and (*ii*) $\theta = 180°$. (*F*) TR fluorescence analysis of (18:1) PC vesicles incubated with (20:1) TAG. (*i*) Confocal slice that gave the longest perimeters for the vesicle with the two membrane regions at the bottom and the unilamellar vesicle in the top right-hand corner. (*ii*) GUV, DB, and SB membrane regions. (*iii*) Distribution of the TR fluorescence intensity for each region. (*G* and *H*) Rupture of a (16:1) PC vesicle with (20:1) TAG droplets. (*G*) Images (*i*) before

(legend continued on next page)

θ–$(S_d/S_s)^{1/2}$ relationships obtained for different TAGs (*circles*, *triangles*, *squares*, and *crosses*) were similar. We therefore plotted all the data for each PC in a single plot. The results revealed that $(S_d/S_s)^{1/2} \geq 1$.

DISCUSSION

Droplet binding to the outer and inner leaflets of GUVs

GUVs bound spherical droplets predominantly to their outer leaflets rather than to their inner leaflets (Fig. 2 *B*). First, we consider the conditions that resulted in spherical droplets. Previous studies have shown that the droplet shape and location are determined by the balance between the bilayer tension and the monolayer tensions on the droplet surface (10,34,35,37). Droplets become spherical at low bilayer tension (10). The bilayer tension arises owing to the osmotic pressure across the bilayer (38). In our study, the osmolarity of the sucrose solution of the initial GUV samples was 21 mOsm/L higher than that of the final sucrose-glucose solution in which the GUVs were suspended (see materials and methods). When a GUV is added to a hypotonic solution, water penetrates the vesicle and therefore the bilayer area expands. Consequently, bilayer tension develops. When the osmolarity difference is very small at the beginning, the vesicle continues to swell until the osmotic pressure is balanced by the Laplace pressure (38,39). However, when the initial osmolarity difference is not small, vesicle swelling leads to the formation of transient pores, resulting in vesicle shrinkage (38,40). In our study, the initial osmolarity difference across the bilayers was high enough to induce pore formation (38). Thus, the bilayer tension varied between different GUVs owing to the leakage of the luminal solution through transient pores. Therefore, the droplet shape likely became spherical when the bilayer tension in the GUV was low.

We next consider the processes through which spherical droplets bound to only one leaflet. A previous study using micro-aspiration reported that a spherical droplet buds toward the monolayer leaflet with a lower monolayer tension (i.e., a higher PC surface density) than the other leaflet (10). Because we did not add external forces to stretch or compress either of the two GUV leaflets, they had an almost equal monolayer tension (i.e., an equal PC surface density) before droplet incorporation. Thus, excess PC lipids that surrounded a spherical droplet to attach it to one leaflet must have been supplied from the outside or inside of the host GUV before or after droplet incorporation. When we mixed a GUV sample with a TAG suspension, the droplets probably adsorbed PC molecules. When a droplet is coated with enough PC lipids, its surface monolayer will link to the outer leaflet of the GUV. Excess PC lipids may also be provided after drop incorporation in the GUV. If a droplet is not sufficiently covered with PC lipids or if the bilayer tension of the GUV is high, the droplet will be incorporated in the intermonolayer space of the GUV. However, if vesicles outside the GUV are adsorbed to the outer surface of the embedded droplet, the droplet will be pushed toward the outer leaflet. By contrast, droplet binding to the inner leaflets will occur through the adsorption of excess PC lipids enclosed in the GUVs. When the GUV contains many vesicles in its lumen, internal vesicles will adsorb to the interior surface of the incorporated droplet, resulting in the droplet being pulled into the luminal side.

When a spherical droplet is incorporated in a low-tension GUV, the size ratio between the droplet and the GUV may influence the droplet incorporation. When a droplet enters a GUV bilayer, some water is removed from the vesicle interior. The excluded water volume is the same as the luminal-side volume in the droplet. The bilayer is permeable only to water (41) until the first bilayer pore transiently opens. Thus, the osmotic pressure from the luminal solution increases during droplet migration toward the vesicle center. Because the increasing osmotic pressure pushes back the droplet, a large droplet relative to the GUV may associate more frequently with the outer leaflet than the inner leaflet.

Structures of vesicles consisting of single-bilayer and double-bilayer regions

The two bilayers stacked in double-bilayer regions may have been bridged by a nonlamellar structure (Fig. 5 *A*i) because they appeared to have the same membrane curvature in the fluorescence images. A plausible bridging structure is stalks, which are an intermediate membrane structure that forms at the onset of vesicle fusion or lamellar-to-hexagonal phase transition (42,43). The stalk structure has a hydrophobic space (Fig. 5 *A*(i), *blue*), which previously has been referred to as a void or an interstice (42–48). The void is actually not vacant and, in the absence of hydrophobic additives, acyl chain tilting and monolayer bending occur to fill this imaginary void with lipid acyl chains (49). The void regions are energetically unstable (42) and can be stabilized by including oil molecules such as alkanes (43–48). Previous studies have shown that diacylglycerol promotes vesicle fusion and non-lamellar phase formation (46,50). A recent study has reported that diacylglycerol

and (*ii*) after rupture constructed from 25 confocal slices with an interslice distance of 0.46–0.49 μm. (*H*) Several slices in the three-dimensional image in (*G*(*ii*)). The values in the images indicate the distances from the glass surface. (*I*) Time-series data for an (18:1) PC vesicle with (18:1) TAG droplets. (*J*) Dependence of the yield of vesicles consisting of unilamellar and bilamellar segments on the PC-TAG pairing. The symbols N_{sd} and N_g denote the numbers of vesicles with two membrane regions and droplet-incorporating unilamellar vesicles, respectively ($n = 3$–7). The data are represented as the mean $\pm$ SE. To see this figure in color, go online.

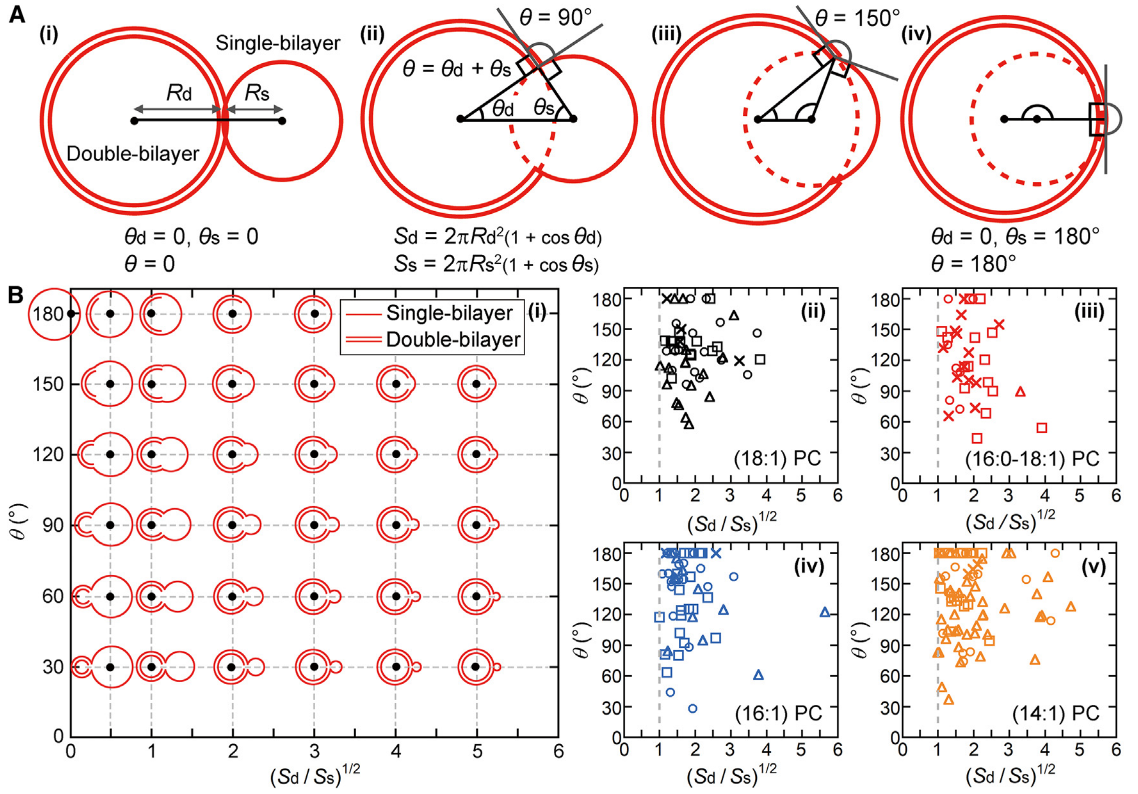

FIGURE 4 Characterization of vesicles consisting of single-bilayer and double-bilayer spherical segments. (*A*) Shape description using the radii of the single-bilayer (R_s) and double-bilayer (R_d) segments, the surface areas of the single-bilayer (S_s) and double-bilayer (S_d) segments, and the intersection angle (θ). θ_s is the angle between the line connecting the sphere centers and the line connecting the single-bilayer sphere center and a sphere intersection point. θ_d is similarly defined for the double-bilayer segment. The sum of θ_s and θ_d equals θ. (*i*)–(*iv*) Vesicle shapes with different θ values obtained with $R_d/R_s = 1.5$ and the constant bilayer surface area. (*B*) Characterization of the vesicle shape. (*i*) Shapes with various $(S_d/S_s)^{1/2}$ and θ values (*black points*) calculated while keeping the total bilayer area constant. Dependence of θ on $(S_d/S_s)^{1/2}$ for (*ii*) (18:1) PC, (*iii*) (16:0–18:1) PC, (*iv*) (16:1) PC, and (*v*) (14:1) PC vesicles incubated with (20:1) TAG (*circles*), (18:1) TAG (*triangles*), (16:1) TAG (*squares*), and (14:1) TAG (*crosses*). To see this figure in color, go online.

greatly stabilizes a stalk structure by accumulating in the void space (47). TAGs are more apolar than diacylglycerols, and have been reported to promote lamellar-to-hexagonal phase transitions (50). We therefore surmise that TAG molecules were likely partitioned in the voids in our experimental system. If the TAG molecules further aggregate in the void, a droplet will emerge (Fig. 5 *A*(*ii*)). This could be the mechanism of droplet binding to the double-bilayer regions. Our data showed that most vesicles (81.1%) anchored TAG droplets to the double-bilayer regions.

Nearly all vesicles (99.5%) bound TAG droplets along the membrane-segment boundary. These droplets will grow similarly to those on the double-bilayer regions. There is a void region along the three-bilayer junction (Fig. 3 *E*, *blue*). If TAG molecules are partitioned in this region, a TAG droplet will grow via further TAG aggregation (Fig. 5 *B*).

Induction of bilayer spontaneous curvature by asymmetric binding of TAG droplets

When a spherical unilamellar vesicle changes into a vesicle consisting of single-bilayer and double-bilayer regions, the luminal volume decreases. In a hypertonic solution, GUVs may undergo such a shape transition because water molecules leave the vesicle lumen across the bilayer, resulting in a smaller luminal volume. However, the sucrose solution used to form the GUVs had a higher osmolarity (358 mOsm/L) than the sucrose-glucose mixture used to suspend the vesicles for fluorescence imaging (337 mOsm/L). This osmolarity difference promoted vesicle swelling and therefore precluded the formation of vesicles with single-bilayer and double-bilayer segments. Thus, there is likely to be a different driving force for membrane separation into the unilamellar and bilamellar regions.

The vesicles consisting of single-bilayer and double-bilayer regions had larger bilayer curvature than the unilamellar vesicles with the same bilayer surface area. In addition, line tension arose along the region boundary. Thus, in the absence of bilayer spontaneous curvature, the formation of vesicles with the two membrane regions was energetically disfavored owing to the increased bending energy and the newly generated line tension energy. Nevertheless, such vesicles were a main vesicle structure for many of the PC-TAG pairs (Fig. 3 *J*). Thus, we expected that bilayer

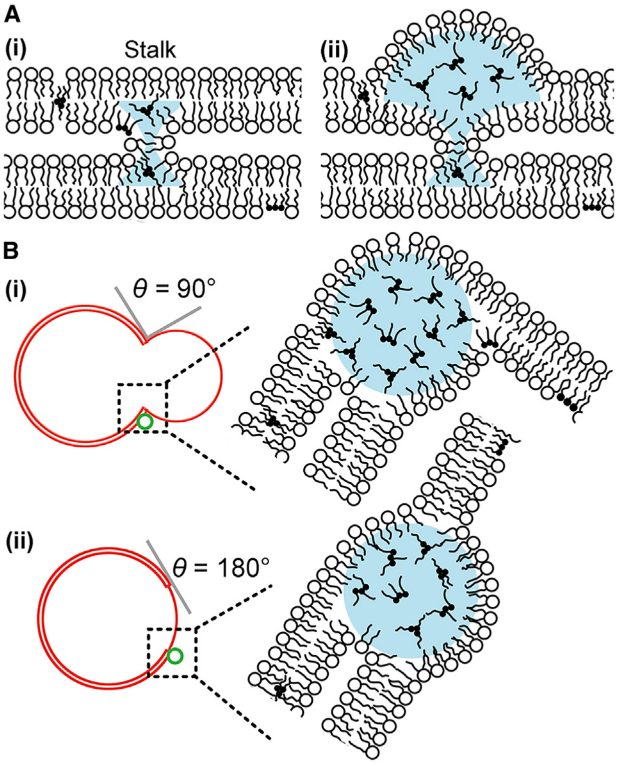

FIGURE 5 Schematic illustrations of the droplet formation processes (*A*) on a double-bilayer region and (*B*) along a region boundary with (*i*) $\theta = 90^\circ$ and (*ii*) $\theta = 180^\circ$. The hydrophobic void in the stalk (*A*(*i*)) or three-bilayer junction (Fig. 3 *E*) can provide a site for droplet growth. To see this figure in color, go online.

spontaneous curvature occurred after droplet incorporation. The spontaneous curvature occurs owing to transbilayer asymmetry (12). Our results showed that GUVs often had an asymmetric surface density of spherical droplets across the bilayers (Fig. 2 *B*) and that vesicles consisting of single-bilayer and double-bilayer regions anchored the droplets to the outermost membrane surfaces, which were exposed to the external solution. We therefore considered that the asymmetric binding of spherical TAG droplets induced bilayer spontaneous curvature.

Bilayer spontaneous curvature is defined differently from monolayer spontaneous curvature (51–53). The former gives zero bilayer-bending energy, whereas the latter gives zero monolayer-bending energy. When the monolayer surrounding a droplet associates with one monolayer leaflet in a flat bilayer (Fig. 6 *A*), the contact region between the droplet and the bilayer has large monolayer curvature. PC lipids have near-zero monolayer spontaneous curvature and therefore favor flat monolayer structures (54–56). Thus, the highly curved PC monolayer in the contact region is unstable. We therefore expect that the flat bilayer with the droplet (Fig. 6 *A*) will bend away from the droplet (Fig. 6 *B*) rather than toward it (Fig. 6 *C*) to reduce the monolayer bending in the droplet contact region. If the droplet surface densities on both leaflets are similar, the global bilayer

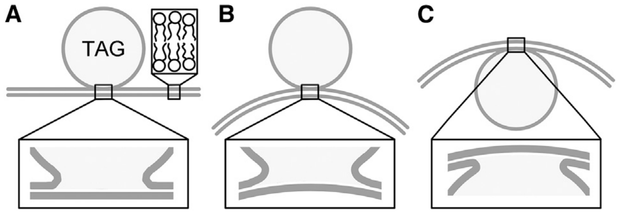

FIGURE 6 Schematic illustrations of a TAG droplet bound to (*A*) a bilayer with zero curvature and (*B* and *C*) bilayers with curvature of opposite signs.

spontaneous curvature disappears. However, droplet binding was often asymmetric across the bilayers (Fig. 2). We therefore expected bilayer spontaneous curvature. When the droplet anchors to the bilayer via a thin neck structure that has a diameter comparable with the bilayer thickness, changes of the bilayer curvature on a micrometer scale do not significantly affect the monolayer bending energy in the neck region. We therefore expect that the neck diameter was larger than the bilayer thickness (Fig. 6). In the following section, we calculate the bending energy by considering the bilayer sheet as the membrane unit. A droplet bound to one leaflet exerts a force that bends the bilayer away from the droplet. The effect of this force is expressed as the bilayer spontaneous curvature.

A recent study reported the formation of dumbbell-like vesicles composed of a single-bilayer segment and a double-bilayer segment after the binding of actin networks only to the inner leaflets of GUVs (57). Notably, in that study, protein binding was asymmetric across the bilayers.

Energy calculation of vesicles consisting of single-bilayer and double-bilayer regions

The bilayer bending energy (G_b) is expressed as (12)

$$G_b = \int dA \frac{\kappa}{2}(C_1 + C_2 - 2C_0)^2, \qquad \text{(Equation 1)}$$

where A is the bilayer surface area, κ is the effective bilayer bending rigidity, C_0 is the bilayer spontaneous curvature, and C_1 and C_2 are the principal curvatures of the bilayer, which are the inverse of the principal radii. The coefficient 2 before C_0 is added such that the bending energy disappears when the mean curvature $(C_1 + C_2)/2$ becomes C_0 (58). In general, the total free energy of a closed vesicle with no domain boundary is the sum of the bending energy and the stretching energy, which varies with the bilayer surface area (58). However, the stretching energy term was omitted because we compared the energies between membrane structures with the same bilayer surface area, as described below.

We calculated the energy difference (ΔG) between a spherical unilamellar vesicle with radius R_0 and a vesicle that had the same bilayer surface area ($4\pi R_0^2$) and consisted of single-bilayer and double-bilayer membrane segments.

We hypothesized that the number and volume of droplets were so small that the effect of the droplets on the energy was negligible. We assumed the same C_0 value for both component bilayers in the double-bilayer region because the two bilayers appeared to have the same curvature in the fluorescence images. From Eq. 1, the bending energy of the unilamellar vesicle ($G_{b,\,0}$) is expressed as

$$G_{b,0} = 8\pi\kappa\left(1 - 2R_0C_0 + {R_0}^2{C_0}^2\right). \qquad \text{(Equation 2)}$$

For the vesicle consisting of spherical segments with radii of $R_d \equiv r_dR_0$ and $R_s \equiv r_sR_0$, the bending energy ($G_{b,\,sd}$) is expressed as

$$G_{b,sd} = 8\pi\kappa\left\{(1+\cos\theta_d)\left(1 - 2r_dR_0C_0 + {r_d}^2{R_0}^2{C_0}^2\right) + \frac{1}{2}(1+\cos\theta_s)\left(1 - 2r_sR_0C_0 + {r_s}^2{R_0}^2{C_0}^2\right)\right\}. \qquad \text{(Equation 3)}$$

Additional energy ($G_{l,\,sd}$) is generated along the membrane segment boundary owing to the line tension (σ). This contribution is expressed as

$$G_{l,sd} = 8\pi\kappa\beta r_d \sin\theta_d, \qquad \text{(Equation 4)}$$

where

$$\beta \equiv \frac{\sigma R_0}{4\kappa}. \qquad \text{(Equation 5)}$$

Because the bilayer surface area is constant, we obtain the following relationship:

$${r_d}^2(1+\cos\theta_d) + \frac{1}{2}{r_s}^2(1+\cos\theta_s) = 1. \qquad \text{(Equation 6)}$$

Using Eqs. 2, 3, 4, 5, and 6, the energy difference between the unilamellar vesicle and the vesicle with the unilamellar and bilamellar regions becomes

$$\frac{\Delta G}{8\pi\kappa} = \frac{1}{8\pi\kappa}(G_{b,sd} + G_{l,sd} - G_{b,0}) = \cos\theta_d + \frac{1}{2}(1+\cos\theta_s) + R_0C_0[2 - 2r_d(1+\cos\theta_d) - r_s(1+\cos\theta_s)] + \beta r_d \sin\theta_d. \qquad \text{(Equation 7)}$$

We calculated $\Delta G/8\pi\kappa$ with different R_0C_0 and β values. We first examined the limits for R_0C_0 and β using the data obtained for vesicles with $\theta = 180°$ (Fig. 3 *E*(*ii*)). The vesicles with $\theta = 180°$ always had $(S_d/S_s)^{1/2} > 1$ (Fig. 4 *B*). Therefore, we assumed that $\Delta G > 0$ for $(S_d/S_s)^{1/2} < 1$ and $\Delta G \leq 0$ for $(S_d/S_s)^{1/2} \geq 1$ at $\theta = 180°$ to determine possible R_0C_0 and β values. The assumed conditions are satisfied when the R_0C_0 and β values are on the black line in Fig. 7 *A*. We therefore used the R_0C_0 and β values on, or close to, the black line. In the gray region above the black line, $(S_d/S_s)^{1/2} < 1$ gives $\Delta G < 0$. Because we did not observe any vesicles with $(S_d/S_s)^{1/2} < 1$, the R_0C_0 and β values were considered not to be in the gray region. On the red line, all $(S_d/S_s)^{1/2}$ values resulted in $\Delta G \geq 0$. As R_0C_0 increases from a point on the red line along a fixed β value to the point on the black line, the range of $(S_d/S_s)^{1/2}$ values that give $\Delta G \leq 0$ becomes broader as follows. The maximum $(S_d/S_s)^{1/2}$ value that gives $\Delta G \leq 0$ is infinite (i.e., the bilamellar vesicle) at any R_0C_0 value, whereas the minimum $(S_d/S_s)^{1/2}$ value decreases from infinity to 1 with increasing R_0C_0.

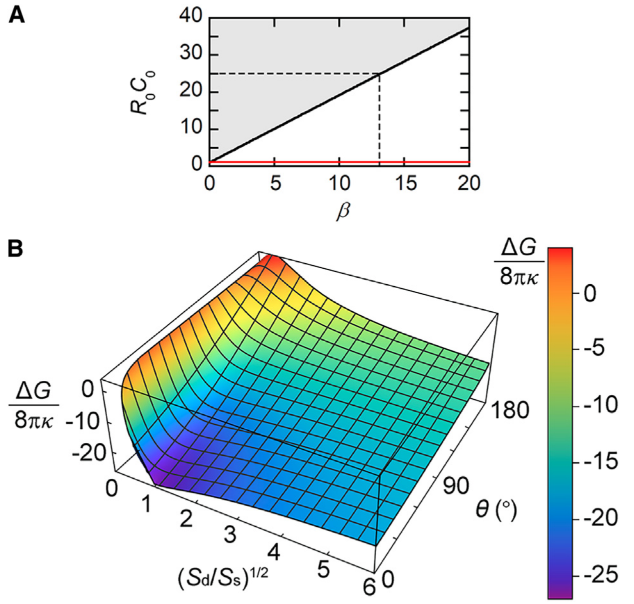

FIGURE 7 Calculated energy difference (ΔG) between a unilamellar vesicle with radius R_0 and a vesicle with a bilayer surface area of $4\pi{R_0}^2$ that consisted of single-bilayer and double-bilayer regions. (*A*) Possible R_0C_0 and β values. We used R_0C_0 and β values on or near the black line with $\beta < 13$ (*dashed lines*). (*B*) Dependence of $\Delta G/8\pi\kappa$ on $(S_d/S_s)^{1/2}$ and θ obtained with $R_0C_0 = 19$ and $\beta = 10$. To see this figure in color, go online.

There is an additional limit for R_0C_0 and β. Previous studies have shown that, when $1/|C_0|$ is small (<200 nm), bilayer tubules form (18,19,59–61). Because we did not observe any tubular vesicles, we assumed that $1/C_0 > 200$ nm. The R_0 values estimated from the fluorescence images were ~5 μm (Table S2). We therefore obtained $R_0C_0 < 25$. A R_0C_0 value of 25 on the black line in Fig. 7 *A* gives $\beta \approx 13$ (*dashed lines*). Thus, we assumed that $\beta < 13$.

The estimation of $\beta < 13$ ($\beta = \sigma R_0/4\kappa$, $R_0 \approx 5$ μm) was consistent with the β limit estimated from literature values of σ and κ. A previous simulation study showed that the line tension along a three-bilayer junction is approximately one-third of the line tension generated along the edge of a bilayer pore (62). In this study, the voids at the three-bilayer junctions were likely occupied by TAG molecules (Fig. 3 *E*, *blue regions*). The line tension would therefore decrease

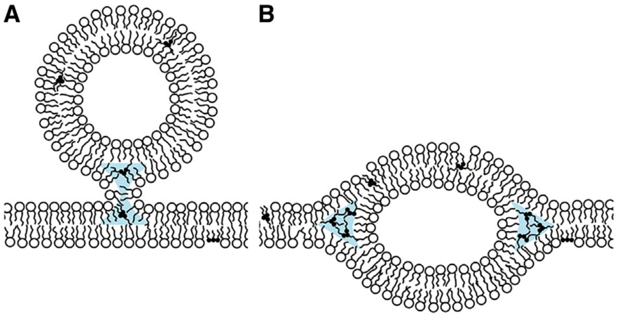

FIGURE 8 The formation of a double-bilayer area in the GUV membrane (*B*) via vesicle binding (*A*). To see this figure in color, go online.

further than for the three-bilayer junction without TAG. Thus, using the line tension value of 20.7 pN reported for the pore edge in (18:1) PC bilayers (63), we obtained $\sigma < 7$ pN. We then estimated the κ value as follows. A previous study found that the bending rigidity of (16:0–18:1) PC bilayers decreased by ~50% after mixing with (18:1) TAG (64). We therefore estimated that $\kappa \approx 0.5 \times 10^{-19}$ J using the bending rigidity reported for PC bilayers (~1×10^{-19} J) (65). These estimates gave $\beta < 175$, which included the estimated region of $\beta < 13$.

We calculated the $\Delta G/8\pi\kappa$ values using Eq. 7. We used the β and R_0C_0 values on or near the black line in Fig. 7 *A* for $\beta < 13$. Representative data (Fig. 7 *B*, $\beta = 10$ and $R_0C_0 = 19$) showed that there was a sharp drop in the energy difference as $(S_d/S_s)^{1/2}$ increased from 0 to 1. For $(S_d/S_s)^{1/2} > 1$, the absolute values of the energy difference tended to decrease with increasing θ or $(S_d/S_s)^{1/2}$. The energy landscapes were consistent with the characteristics observed in the experiments (Fig. 4 *B*). First, for all vesicles, $(S_d/S_s)^{1/2} \geq 1$. Second, vesicles with larger $(S_d/S_s)^{1/2}$ values tended to have smaller θ values. Thus, the calculated results supported the existence of bilayer spontaneous curvature.

Vesicle shape-transition mechanisms

We now consider the process where a unilamellar vesicle deforms into a vesicle consisting of single-bilayer and double-bilayer regions. When the unilamellar vesicle ($(S_d/S_s)^{1/2} = 0$, $\theta = 180°$) changes to a lower-energy structure by moving over the calculated potential surface (Fig. 7 *B*), a negligibly small bilamellar area must form first (Fig. 8). This step can initiate with the binding of a small vesicle present inside or outside the GUV (Fig. 8 *A*). After the bound vesicle dislocates to the center of the GUV bilayer (Fig. 8 *B*), the bilamellar region forms. TAGs promote the formation of non-lamellar phases (50), and therefore may facilitate the proposed mechanism, which involves the formation of non-lamellar structures.

The vesicle that first binds to the GUV bilayer (Fig. 8 *A*) does not need to be small. In real systems, there are no limits on the size of the bound vesicle and on the θ and $(S_d/S_s)^{1/2}$ values of the initial structure that results from vesicle binding. This means that the vesicle shape transition may start from anywhere on the potential surface (Fig. 7 *B*).

CONCLUSIONS

In this study, GUVs bound spherical TAG droplets predominantly to their outer leaflet rather than to their inner leaflet. Thus, the surface density of the spherical TAG droplets became asymmetric across the bilayer membrane. We expect that this transbilayer asymmetry induced bilayer spontaneous curvature, thereby triggering the formation of vesicles consisting of a single-bilayer segment and a double-bilayer segment.

SUPPORTING MATERIAL

Supporting material can be found online at https://doi.org/10.1016/j.bpj.2024.05.030.

AUTHOR CONTRIBUTIONS

The author confirms being the sole contributor of this work and has approved it for publication.

ACKNOWLEDGMENTS

This work was supported by a KAKENHI grant (22K05179), the Japan Society for the Promotion of Science. We thank Edanz (https://jp.edanz.com/ac) for editing a draft of this manuscript.

DECLARATION OF INTERESTS

The authors declare no competing interests.

REFERENCES

1. Olzmann, J. A., and P. Carvalho. 2019. Dynamics and functions of lipid droplets. *Nat. Rev. Mol. Cell Biol.* 20:137–155.
2. Walther, T. C., and R. V. Farese, Jr. 2012. Lipid droplets and cellular lipid metabolism. *Annu. Rev. Biochem.* 81:687–714.
3. Tauchi-Sato, K., S. Ozeki, …, T. Fujimoto. 2002. The surface of lipid droplets is a phospholipid monolayer with a unique fatty acid composition. *J. Biol. Chem.* 277:44507–44512.
4. Small, D. M. 1970. The physical state of lipids of biological importance: Cholesteryl esters, cholesterol, triglyceride. *In* Advances in Experimental Medicine and Biology, Vol 7. Surface Chemistry of Biological Systems. M. Blank, ed Springer, Boston, MA, pp. 55–83.
5. Shimobayashi, S. F., and Y. Ohsaki. 2019. Universal phase behaviors of intracellular lipid droplets. *Proc. Natl. Acad. Sci. USA.* 116:25440–25445.
6. Kataoka-Hamai, C., and K. Kawakami. 2021. Determining the dependence of interfacial tension on molecular area for phospholipid monolayers formed at silicone oil–water and tricaprylin–water interfaces by vesicle fusion. *Langmuir.* 37:7527–7535.
7. Mitsche, M. A., L. Wang, and D. M. Small. 2010. Adsorption of egg phosphatidylcholine to an air/water and triolein/water bubble interface: Use of the 2-dimensional phase rule to estimate the surface

composition of a phospholipid/triolein/water surface as a function of surface pressure. *J. Phys. Chem. B.* 114:3276–3284.
8. Kataoka-Hamai, C., and K. Kawakami. 2023. Ostwald ripening of triacylglycerol droplets embedded in glass-supported phospholipid bilayers. *Langmuir.* 39:10001–10010.
9. Salo, V. T., S. Li, …, E. Ikonen. 2019. Seipin facilitates triglyceride flow to lipid droplet and counteracts droplet ripening via endoplasmic reticulum contact. *Dev. Cell.* 50:478–493.e9.
10. Chorlay, A., and A. R. Thiam. 2018. An asymmetry in monolayer tension regulates lipid droplet budding direction. *Biophys. J.* 114:631–640.
11. Chorlay, A., A. Santinho, and A. R. Thiam. 2020. Making droplet-embedded vesicles to model cellular lipid droplets. *STAR Protoc.* 1:100116.
12. Hossein, A., and M. Deserno. 2020. Spontaneous curvature, differential stress, and bending modulus of asymmetric lipid membranes. *Biophys. J.* 118:624–642.
13. Nikolov, V., R. Lipowsky, and R. Dimova. 2007. Behavior of giant vesicles with anchored DNA molecules. *Biophys. J.* 92:4356–4368.
14. Stachowiak, J. C., C. C. Hayden, and D. Y. Sasaki. 2010. Steric confinement of proteins on lipid membranes can drive curvature and tubulation. *Proc. Natl. Acad. Sci. USA.* 107:7781–7786.
15. Ayton, G. S., P. D. Blood, and G. A. Voth. 2007. Membrane remodeling from N-BAR domain interactions: Insights from multi-scale simulation. *Biophys. J.* 92:3595–3602.
16. Peter, B. J., H. M. Kent, …, H. T. McMahon. 2004. BAR domains as sensors of membrane curvature: The amphiphysin BAR structure. *Science.* 303:495–499.
17. Graber, Z. T., Z. Shi, and T. Baumgart. 2017. Cations induce shape remodeling of negatively charged phospholipid membranes. *Phys. Chem. Chem. Phys.* 19:15285–15295.
18. Karimi, M., J. Steinkühler, …, R. Dimova. 2018. Asymmetric ionic conditions generate large membrane curvatures. *Nano Lett.* 18:7816–7821.
19. Dasgupta, R., M. S. Miettinen, …, R. Dimova. 2018. The glycolipid GM1 reshapes asymmetric biomembranes and giant vesicles by curvature generation. *Proc. Natl. Acad. Sci. USA.* 115:5756–5761.
20. Heuvingh, J., and S. Bonneau. 2009. Asymmetric oxidation of giant vesicles triggers curvature-associated shape transition and permeabilization. *Biophys. J.* 97:2904–2912.
21. Bhatia, T., S. Christ, …, R. Lipowsky. 2020. Simple sugars shape giant vesicles into multispheres with many membrane necks. *Soft Matter.* 16:1246–1258.
22. Walther, T. C., J. Chung, and R. V. Farese. 2017. Lipid droplet biogenesis. *Annu. Rev. Cell Dev. Biol.* 33:491–510.
23. Nixon-Abell, J., C. J. Obara, …, J. Lippincott-Schwartz. 2016. Increased spatiotemporal resolution reveals highly dynamic dense tubular matrices in the peripheral ER. *Science.* 354:aaf3928.
24. Shibata, Y., T. Shemesh, …, T. A. Rapoport. 2010. Mechanisms determining the morphology of the peripheral ER. *Cell.* 143:774–788.
25. Kassan, A., A. Herms, …, A. Pol. 2013. Acyl-CoA synthetase 3 promotes lipid droplet biogenesis in ER microdomains. *J. Cell Biol.* 203:985–1001.
26. Santinho, A., V. T. Salo, …, A. R. Thiam. 2020. Membrane curvature catalyzes lipid droplet assembly. *Curr. Biol.* 30:2481–2494.e6.
27. Chen, P. S., T. Y. Toribara, and H. Warner. 1956. Microdetermination of phosphorus. *Anal. Chem.* 28:1756–1758.
28. Angelova, M. I., S. Soléau, …, P. Bothorel. 1992. Preparation of giant vesicles by external AC electric vields. Kinetics and applications. *Prog. Colloid Polym. Sci.* 89:127–131.
29. Kataoka-Hamai, C., and K. Kawakami. 2021. Domain sorting in giant unilamellar vesicles adsorbed on glass. *Langmuir.* 37:1082–1088.
30. Witkowska, A., L. Jablonski, and R. Jahn. 2018. A convenient protocol for generating giant unilamellar vesicles containing SNARE proteins using electroformation. *Sci. Rep.* 8:9422.
31. Abramoff, M. D., P. J. Magalhaes, and S. J. Ram. 2004. Image processing with ImageJ. *Biophot. Int.* 11:36–42.
32. Rasband, W. S. 1997–2018. ImageJ. National Institutes of Health, Bethesda, Maryland. https://imagej.nih.gov/ij/.
33. Schneider, C. A., W. S. Rasband, and K. W. Eliceiri. 2012. NIH Image to ImageJ: 25 years of image analysis. *Nat. Methods.* 9:671–675.
34. Santinho, A., A. Chorlay, …, A. R. Thiam. 2021. Fat inclusions strongly alter membrane mechanics. *Biophys. J.* 120:607–617.
35. Chorlay, A., L. Forêt, and A. R. Thiam. 2021. Origin of gradients in lipid density and surface tension between connected lipid droplet and bilayer. *Biophys. J.* 120:5491–5503.
36. Akashi, K., H. Miyata, …, K. Kinosita. 1996. Preparation of giant liposomes in physiological conditions and their characterization under an optical microscope. *Biophys. J.* 71:3242–3250.
37. Retter, U., and D. Vollhardt. 1993. Formation of lenticular nuclei from an insoluble monolayer at the air/water interface: A model. *Langmuir.* 9:2478–2480.
38. Alam Shibly, S. U., C. Ghatak, …, M. Yamazaki. 2016. Experimental estimation of membrane tension induced by osmotic pressure. *Biophys. J.* 111:2190–2201.
39. Levin, Y., and M. A. Idiart. 2004. Pore dynamics of osmotically stressed vesicles. *Phys. A: Stat. Mech. Appl.* 331:571–578.
40. Saha, S. K., S. U. Alam Shibly, and M. Yamazaki. 2020. Membrane tension in negatively charged lipid bilayers in a buffer under osmotic pressure. *J. Phys. Chem. B.* 124:5588–5599.
41. Olbrich, K., W. Rawicz, …, E. Evans. 2000. Water permeability and mechanical strength of polyunsaturated lipid bilayers. *Biophys. J.* 79:321–327.
42. Siegel, D. P. 1999. The modified stalk mechanism of lamellar/inverted phase transitions and its implications for membrane fusion. *Biophys. J.* 76:291–313.
43. Siegel, D. P. 1993. Energetics of intermediates in membrane fusion: Comparison of stalk and inverted micellar intermediate mechanisms. *Biophys. J.* 65:2124–2140.
44. Malinin, V. S., and B. R. Lentz. 2004. Energetics of vesicle fusion intermediates: Comparison of calculations with observed effects of osmotic and curvature stresses. *Biophys. J.* 86:2951–2964.
45. Siegel, D. P., J. Banschbach, and P. L. Yeagle. 1989. Stabilization of H_{II} phases by low levels of diglycerides and alkanes: An NMR, calorimetric, and X-ray diffraction study. *Biochemistry.* 28:5010–5019.
46. Basáñez, G., J. L. Nieva, …, F. M. Goñi. 1996. Diacylglycerol and the promotion of lamellar-hexagonal and lamellar-isotropic phase transitions in lipids: Implications for membrane fusion. *Biophys. J.* 70:2299–2306.
47. Poojari, C. S., K. C. Scherer, and J. S. Hub. 2021. Free energies of membrane stalk formation from a lipidomics perspective. *Nat. Commun.* 12:6594.
48. Walter, A., P. L. Yeagle, and D. P. Siegel. 1994. Diacylglycerol and hexadecane increase divalent cation-induced lipid mixing rates between phosphatidylserine large unilamellar vesicles. *Biophys. J.* 66:366–376.
49. Kozlovsky, Y., L. V. Chernomordik, and M. M. Kozlov. 2002. Lipid intermediates in membrane fusion: Formation, structure, and decay of hemifusion diaphragm. *Biophys. J.* 83:2634–2651.
50. Epand, R. M., R. F. Epand, and C. R. Lancaster. 1988. Modulation of the bilayer to hexagonal phase transition of phosphatidylethanolamines by acylglycerols. *Biochim. Biophys. Acta.* 945:161–166.
51. Dimova, R. 2019. Giant vesicles and their use in assays for assessing membrane phase state, curvature, mechanics, and electrical properties. *Annu. Rev. Biophys.* 48:93–119.
52. Fuller, N., and R. P. Rand. 2001. The influence of lysolipids on the spontaneous curvature and bending elasticity of phospholipid membranes. *Biophys. J.* 81:243–254.

53. Hamai, C., T. Yang, …, S. M. Musser. 2006. Effect of average phospholipid curvature on supported bilayer formation on glass by vesicle fusion. *Biophys. J.* 90:1241–1248.

54. Kollmitzer, B., P. Heftberger, …, G. Pabst. 2013. Monolayer spontaneous curvature of raft-forming membrane lipids. *Soft Matter.* 9:10877–10884.

55. Orsi, M., J. Michel, and J. W. Essex. 2010. Coarse-grain modelling of DMPC and DOPC lipid bilayers. *J. Phys. Condens. Matter.* 22:155106.

56. Szule, J. A., N. L. Fuller, and R. P. Rand. 2002. The effects of acyl chain length and saturation of diacylglycerols and phosphatidylcholines on membrane monolayer curvature. *Biophys. J.* 83:977–984.

57. Baldauf, L., F. Frey, …, G. H. Koenderink. 2023. Branched actin cortices reconstituted in vesicles sense membrane curvature. *Biophys. J.* 122:2311–2324.

58. Lipowsky, R. 2022. Remodeling of membrane shape and topology by curvature elasticity and membrane tension. *Adv. Biol.* 6:2101020.

59. Ali Doosti, B., W. Pezeshkian, …, T. Lobovkina. 2017. Membrane tubulation in lipid vesicles triggered by the local application of calcium ions. *Langmuir.* 33:11010–11017.

60. Heinrich, M. C., B. R. Capraro, …, T. Baumgart. 2010. Quantifying Membrane Curvature Generation of *Drosophila* Amphiphysin N-BAR Domains. *J. Phys. Chem. Lett.* 1:3401–3406.

61. Shukla, S., R. Jin, …, T. Baumgart. 2019. PIP2 reshapes membranes through asymmetric desorption. *Biophys. J.* 117:962–974.

62. Risselada, H. J., G. Marelli, …, M. Müller. 2012. Line-tension controlled mechanism for influenza fusion. *PLoS One.* 7:e38302.

63. Karatekin, E., O. Sandre, …, F. Brochard-Wyart. 2003. Cascades of transient pores in giant vesicles: Line tension and transport. *Biophys. J.* 84:1734–1749.

64. Pakkanen, K. I., L. Duelund, …, J. H. Ipsen. 2011. Mechanics and dynamics of triglyceride-phospholipid model membranes: Implications for cellular properties and function. *Biochim. Biophys. Acta.* 1808:1947–1956.

65. Rawicz, W., K. C. Olbrich, …, E. Evans. 2000. Effect of chain length and unsaturation on elasticity of lipid bilayers. *Biophys. J.* 79:328–339.

Biophysical Journal, Volume *123*

Supplemental information

Triacylglycerol-droplet-induced bilayer spontaneous curvature in giant unilamellar vesicles

Chiho Kataoka-Hamai

Table S1. Vesicle radii (R_v) of unilamellar vesicles incorporating TAG droplets.

PC	TAG	R_v (± standard error) (μm)	
18:1	14:1	3.79 (± 0.26)	(N = 30)
	16:1	3.50 (± 0.18)	(N = 18)
	18:1	3.63 (± 0.23)	(N = 19)
	20:1	3.76 (± 0.23)	(N = 24)
16:0–18:1	14:1	3.31 (± 0.22)	(N = 19)
	16:1	3.11 (± 0.23)	(N = 23)
	18:1	3.36 (± 0.31)	(N = 21)
	20:1	4.02 (± 0.66)	(N = 9)
16:1	14:1	3.16 (± 0.12)	(N = 35)
	16:1	3.12 (± 0.14)	(N = 23)
	18:1	2.80 (± 0.24)	(N = 16)
	20:1	2.75 (± 0.16)	(N = 31)
14:1	14:1	4.28 (± 0.33)	(N = 31)
	16:1	3.82 (± 0.60)	(N = 11)
	18:1	4.79 (± 0.37)	(N = 13)
	20:1	4.22 (± 0.36)	(N = 9)

Table S2. Size parameters (R_0) of the vesicles consisting of single-bilayer and double-bilayer segments. The R_0 values were calculated by assuming that the vesicles had a total bilayer surface area of $4\pi R_0^2$.

PC	TAG	R_0 (± standard error) (μm)	
18:1	14:1	5.82 (± 0.85)	(N = 4)
	16:1	5.53 (± 0.50)	(N = 14)
	18:1	5.99 (± 0.45)	(N = 17)
	20:1	7.27 (± 0.58)	(N = 16)
16:0–18:1	14:1	6.01 (± 0.63)	(N = 13)
	16:1	6.40 (± 0.72)	(N = 15)
	18:1	3.56	(N = 1)
	20:1	5.25 (± 0.57)	(N = 7)
16:1	14:1	3.62 (± 0.07)	(N = 2)
	16:1	4.90 (± 0.34)	(N = 21)
	18:1	5.42 (± 0.41)	(N = 12)
	20:1	4.31 (± 0.30)	(N = 19)
14:1	14:1	5.08 (± 0.80)	(N = 4)
	16:1	5.39 (± 0.32)	(N = 18)
	18:1	7.54 (± 0.47)	(N = 39)
	20:1	5.49 (± 0.34)	(N = 20)
